# Exploring the Applicability of Calorespirometry to Assess Seed Metabolic Stability Upon Temperature Stress Conditions—*Pisum sativum* L. Used as a Case Study

**DOI:** 10.3389/fpls.2022.827117

**Published:** 2022-04-27

**Authors:** Lénia Rodrigues, Amaia Nogales, Lee D. Hansen, Fátima Santos, Ana Elisa Rato, Hélia Cardoso

**Affiliations:** ^1^Mediterranean Institute for Agriculture, Environment and Development, Instituto de Formação e Investigação Avançada, Universidade de Évora, Évora, Portugal; ^2^Linking Landscape, Environment, Agriculture and Food, Instituto Superior de Agronomia, Universidade de Lisboa, Lisbon, Portugal; ^3^Department of Chemistry and Biochemistry, Brigham Young University, Provo, UT, United States; ^4^Centro Nacional de Biotecnología, Unidad de Proteomica, CSIC, Calle Darwin 3, Madrid, Spain; ^5^Mediterranean Institute for Agriculture, Environment and Development, Departamento de Fitotecnia, Escola de Ciências e Tecnologia, Universidade de Évora, Évora, Portugal

**Keywords:** phenotyping, alternative oxidase, germination, metabolism, calorespirometry, *Fabaceae*, pea

## Abstract

The availability of phenotyping tools to assist breeding programs in the selection of high-quality crop seeds is of obvious interest with consequences for both seed producers and consumers. Seed germination involves the activation of several metabolic pathways, such as cellular respiration to provide the required ATP and reducing power. This work tested the applicability of calorespirometry, the simultaneous measurement of heat and CO_2_ rates, as a phenotyping tool to assess seed respiratory properties as a function of temperature. The effect of temperature on seed germination was evaluated after 16 h of seed imbibition by calorespirometric experiments performed in isothermal mode at 15, 20, 25, and 28°C on the seeds of three cultivars of peas (*Pisum sativum* L.) commonly used in conventional agriculture (cvs. ‘Rondo’, ‘Torta de Quebrar’, and ‘Maravilha d’América’). Significant differences in metabolic heat rate and CO_2_ production rate (R_*CO2*_) as well as in the temperature responses of these parameters were found among the three cultivars. A seed germination trial was conducted during the 6 days of imbibition to evaluate the predictive power of the parameters derived from the calorespirometric measurements. The germination trial showed that the optimal germination temperature was 20°C and low germination rates were observed at extreme temperatures (15 or 28°C). The cv. ‘Torta de Quebrar’ showed significantly higher germination in comparison with the other two cultivars at all three temperatures. In comparison with the other two cultivars, ‘Torta de Quebrar’ has the lowest metabolic heat and CO_2_ rates and the smallest temperature dependence of these measured parameters. Additionally, ‘Torta de Quebrar’ has the lowest values of growth rate and carbon use efficiency calculated from the measured variables. These data suggest that calorespirometry is a useful tool for phenotyping physiologic efficiency at different temperatures during early germination stages, and can determine the seeds with the highest resilience to temperature variation, in this case ‘Torta de Quebrar’.

## Introduction

Seeds are central to crop production, human nutrition, and food security, and the complex trait of seed vigor is a key component of the performance of crops (Finch-Savage and Bassel, 2016). Seed vigor, comprised of germination and seedling performance, affects vegetative growth and is frequently related to crop yield (TeKrony and Egli, 1991; Ellis, 1992). Improving seed vigor is therefore a primary objective of the agricultural industry and the seed/breeding companies that support it (Finch-Savage and Bassel, 2016). An increase of 15–20% yield has been reported if high-quality seeds are used (Ambika et al., 2014). Seed performance depends on genetic factors and on the environmental conditions the parent plant was exposed to during the formation and the development of seeds as well as the interaction between genetics and environmental conditions during germination (Bettey et al., 2000; Hampton et al., 2013; Li et al., 2017). Temperature, water potential, and oxygen have been recognized as the main environmental factors affecting germination (Finch-Savage and Bassel, 2016). Additionally, some physiological factors have also been indicated as the modulators of seed germination. Dormancy associated with tegument (seed coat), named as physical dormancy (Baskin and Baskin, 2014), has been highlighted as an endogenous factor with a high effect on seed germination (Ferraz et al., 2019), attributed to the presence of a mechanical obstacle to water and oxygen circulation, and to the presence of hormonal substances with an inhibitor role on germination.

In breeding programs, the availability of a phenotyping tool able to select high-quality seeds, more resilient upon stress factors has an obvious interest. Different phenotyping techniques have been used to assess seed viability, which is defined as the capacity of seed to efficiently germinate and give rise to a seedling with the potential to further develop into a plant. Few methods are currently available for assessing the physiological quality of seed, which influences not only the seed viability but also seedling establishment (seed vigor). Previous reports demonstrated the applicability of ethanol-based methods to assess seed quality and vigor (Taylor et al., 1999; Buckley and Huang, 2011; Kodde et al., 2012). During germination, the progressive depletion of oxygen creates anaerobic conditions consequently leading to the activation of fermentative metabolism, working as the main source of ATP production (Ma et al., 2020). Recently developed methods for assessing physiological quality are based on the measurement of metabolic products, in particular, the quantification of respiratory products [for a review see Dalzielli and Tomlinson (2017)]. Oxygen consumption and CO_2_ production have been used to detect the differences in seed quality from seed or seed-lot respiration (Zhao and Zhong, 2012; Bradford et al., 2013; Patanè and Avola, 2013; Xin et al., 2013; Bello and Bradford, 2016). Dalzielli and Tomlinson (2017) have recently demonstrated a linear relationship between seed viability and metabolic rate determined with flow-through respirometry in the seeds of several plant species. In addition to CO_2_ production rates, plant respiration is linked to the production of metabolic heat, and plant growth rates have been correlated with heat rates (Criddle et al., 1991). Calorespirometry is a rapid method for simultaneously measuring the metabolic heat rate and CO_2_ emission rate of biological samples at different environmental temperatures (Nogales et al., 2013, 2015, 2020). Combining respiratory heat and CO_2_ rates as a function of temperature enables the calculation of carbon use efficiency (the fraction of carbon substrate incorporated into structural biomass, ε) and growth rate as functions of temperature to select the temperature range to which plants are well-adapted (Hansen et al., 2009). This approach has been used as a screening tool to assess metabolic and respiratory changes associated with cell reprogramming events (Arnholdt-Schmitt et al., 2018) that include plant plasticity towards different growing temperatures (Nogales et al., 2013, 2015, 2020; Campos et al., 2016). Most calorespirometric studies have been done with actively growing tissues, such as the cambial tissue taken from carrot taproots (Nogales et al., 2013, 2015), young leaves or shoots taken from seedlings/plantlets (Matheson et al., 2004; Nogales et al., 2020), and somatic *calli* characterized by active cell mitoses (Campos et al., 2016). The previous use of calorespirometry on seeds is limited, but the applicability of calorespirometry for the seed phenotyping of melon is known from Edelstein et al. (2001), and more recently of carrot, chickpea, and pea seeds by Mohanapriya et al. (2019).

For the present research, pea (*Pisum sativum* L.) was selected based on its agronomical interest. This species is a widespread crop across Europe, playing a preponderant role in human nutrition as one of the main sources of dietary plant protein (Dhall, 2017). In addition to the high protein content (23–31% of seed dry matter), pea seeds are nutritious due to minerals, carbohydrates, fiber, and several bioactive compounds, such as polyphenolics, vitamins, saponins, and galactose oligosaccharides (Karkanis et al., 2016). Pea is adapted to a wide range of climatic regimes, from semiarid to temperate maritime, with a germination temperature ranging from 20 to −1.1°C (Raveneau et al., 2011). One of the main goals in pea breeding is to obtain climate-adapted genotypes with increased tolerance to adverse environmental conditions. However, studies on the effects of adverse environmental conditions on this plant species have only been done at the plant level (Hernandez et al., 2000; Allen and Ort, 2001). The present study, focused on the seed phase, has a high interest on exploring the potentiality of calorespirometry to assess metabolic stability during the germination phase when exposed to different environmental temperatures.

## Materials and Methods

### Plant Material

Three cultivars of pea (*P. sativum*) provided by flora Lusitana^®^, commonly used in conventional agriculture were selected for calorespirometric measurements and germination trials: cvs. ‘Rondo’ (lots YF001522 and G2003378), ‘Torta de Quebrar’ (lots FL5581 and FL5719), and ‘Maravilha d’América’ (lots 04528CIT07X and AN3815-7251).

### Characterizing the Imbibition Period by Calorespirometric Measurements

Seed hydration initiates the germination process. Two complementary experiments were conducted with the aim of characterizing the water imbibition period. In the first experiment, the seeds of cvs. ‘Rondo’, ‘Torta de Quebrar’, and ‘Maravilha d’América’ were placed in sterile tap water and immediately introduced into a Multi-Cell Differential Scanning Calorimeter (TA Instruments, Lindon, UT, United States) to monitor heat rates in the isothermal mode at 25°C during 24 h. A single seed was inserted in each 1 cm^3^ ampoule (1 empty ampoule for reference and 3 ampoules for pea seeds) containing 500 μl of distilled sterile water. In the second experiment, seed water uptake (WU) was determined over 48 h by soaking 30 seeds in water at 25°C for 0, 4, 8, 12, 16, 24, and 48 h. The percentage of WU was calculated according to the equation:


(1) $$U\,P=\left[\frac{M\,f-P\,i}{P\,i}\right]\,x\,100$$

where *UP* is the percentage of WU by seeds, *Mf* is the fresh seed mass after soaking, and *Pi* is the dry seed mass before soaking (Silva et al., 2018).

### Evaluation of Calorespirometric Parameters During Germination at Different Temperatures

The effect of temperature on seed metabolism was evaluated by calorespirometry of seeds after 16 h of imbibition in sterile tap water. During imbibition, the seeds of three cultivars (‘Rondo’ lot YF001522, cv. ‘Torta de Quebrar’ lot FL5581, and cv. ‘Maravilha d’América’ lot 04528CIT07X) were kept at 15, 20, 25, and 28°C, and calorespirometric measurements were conducted at the same temperature used for imbibition. Calorespirometric measurements were performed in the isothermal mode. An equilibration time of 5–10 min prior to data collection allowed for stabilization after which heat rate data were collected for another 10–15 min. After the initial heat rate from each seed was recorded, a vial containing 50 μl of 0.4 M NaOH solution was introduced into the ampoules, the heat rate was again recorded after it became stable, and the vial containing the NaOH was then removed from the ampoules. A third measurement of the heat rate was then made. Supplementary Figure 1 shows an example of the raw data collected on each cultivar at 25°C. Raw data were corrected with a constant instrument baseline measured at each temperature with empty ampoules. To evaluate the effect of the lot, the same procedure was repeated for a different seed lot of each cultivar (‘Rondo’ lot G2003378, cv. ‘Torta de Quebrar’ lot FL5719, and cv. ‘Maravilha d’América’ lot AN3815-7251) running the calorimeter under isothermal mode at 25°C. Respiratory heat rate (R_q_) was calculated as the average of the first and third measurements. Respiration CO_2_ rate (R_*CO2*_) was calculated based on the principle that CO_2_ reacts with the NaOH to form carbonate according to the equation: CO_2_ (g) + 2OH^–^ (aq) = CO_3_^2–^ (aq) + H_2_O with an exothermic enthalpy change of -108.5 kJ/mol (Criddle et al., 1990; Hansen et al., 2005). Respiration CO_2_ rate was calculated from the increase in the measured heat rate that happens during the second measurement (Criddle et al., 1990; Hansen et al., 2005).

The values of R_q_ and R_*CO2*_ were used to calculate the R_q_/R_*CO2*_ ratio, structural biomass formation rate (R_*struct_biomass*_), and carbon use efficiency (ε) (Criddle et al., 1991; Hansen et al., 2005). The R*q*/R_*CO2*_ ratio is useful as an indication of efficiency as the amount of heat lost from plant tissue per mole of CO_2_ produced (Hansen et al., 2005). Specific growth rate or structural biomass formation rate (R_*struct_biomass*_) and ε, the substrate carbon conversion efficiency or the fraction of substrate carbon incorporated into new structural biomass, were calculated from R_q_ and R_*CO2*_ values with an enthalpy balance model that considers the heat released by tissue is equal to the sum of heat from catabolic reactions plus heat absorbed by anabolic reactions (Hansen et al., 2002, 2005; Macfarlane et al., 2002; Nogales et al., 2020). The values of R_q_, R_*CO2*_, and R_*struct_biomass*_ were normalized to seed dry mass before imbibition. Carbon use efficiency is a unitless fraction and therefore does not require normalization.

### Evaluation of Temperature Effect on Seed Germination and Seedling Development

The effect of temperature on seed germination was evaluated by a germination trial run for the three cultivars, ‘Rondo’, ‘Torta de Quebrar’, and ‘Maravilha d’América’. The seeds of each cultivar were placed in 3 cm diameter wells in a tray and covered with cotton imbibed with sterile tap water. Seeds were kept permanently wet and incubated in complete darkness and 75% relative humidity at 15, 20, 25, or 28°C. Germination tests were done with 9 replicates of 10 seeds each for a total of 90 seeds per temperature and cultivar (360 seeds per cultivar). Determination of the number of germinated seeds was made daily. A seed was considered germinated when a visible radicle protruded more than 1 mm (Nakasato et al., 2017). Seed germination achieved at each temperature over 6 days (cumulative germination) was calculated using the equation:


(2) Germination(%)=(Numberofgerminatedseeds/TS)x100

where *TS* is the total number of seeds of each cultivar used in the assay. The agronomically acceptable minimum (aam) germination is 70% for pea seeds (Miguel, 1983). The percentage of seeds germinated per day (*PG*) was determined (Silva et al., 2018) according to the equation:


(3) PG(%)=(∑ni/N)x100

where *ni* corresponds to the number of seeds germinated on the day, and *N* is the total number of germinated seeds. Total germination rate (TG) achieved at each temperature is the total of germinated seeds at the end of 6 days of imbibition, and was calculated according to the equation:


(4) $$TG\left(\%\right)=\left(\frac{\sum n\,t}{N}\right)x100$$

where *nt* is the total number of germinated seeds after 6 days of imbibition and *N* is the total number of seeds used in the experiment.

Seeds were considered dead when there was mold growth and consequent seed degradation. The percentage of dead seeds (DS) was calculated according to the equation:


(5) DS(%)=(Numberofdeadseeds/TS)x100

To assess the seedling development at different temperatures, the lengths of primary roots and shoots were measured after 6 days of imbibition. Germination was interpreted according to Weitbrecht et al. (2011), who defined three phases of germination: phase I (early phase) includes the rapid imbibition and the early plateau phase of WU; phase II (middle phase) includes the plateau phase of WU and radicle protrusion; and phase III (later phase) corresponds to the seedling developmental phase, and is also called the post-germinative phase (Ali and Elozeiri, 2017). The control of these three phases of germination has been highlighted as an important factor in successful agricultural production due to its effect on early seedling growth, pre-harvest sprouting/dormancy, and longevity (storability) (Sano et al., 2020).

### Evaluation of Tegument (Physical Inhibition) on Seed Germination Efficiency

To evaluate the effect of the tegument on seed germination of the three cultivars, ‘Rondo’, ‘Torta de Quebrar’, and ‘Maravilha d’América’, intact seeds and seeds scarified following a mechanical procedure by making small scratches with sandpaper, were placed in trays with 3 cm diameter wells, covered with cotton imbibed with sterile tap water and incubated under dark conditions, 75% relative humidity, and temperature of 25°C. Then, 16 h after imbibition, the same period used for calorimetric measurements, the intact seeds were removed from their tegument. At this time point, the radicle length was measured on each seed. Seeds maintaining an intact embryo and endosperm were further kept under the same conditions for 6 days to assess the total germination rate. The same conditions and procedures were followed for seeds that were previously scarified. Both experiments, using intact seeds and scarified seeds, were done with 3 replicates of 10 seeds each for a total of 30 seeds per temperature and cultivar.

### Statistical Analysis

Statistical analyses were performed by SPSS version 22.0. Normality and homoscedasticity were checked for all data and mean comparisons performed by one-way ANOVA followed by Tukey’s HSD test. When the data did not meet the assumptions for performing parametric tests, comparisons were performed by the Kruskal–Wallis nonparametric test. Statistical significance was considered at *p* < 0.05.

## Results

### Characterizing Early Imbibition and Metabolic Activation

**FIGURE 1:**
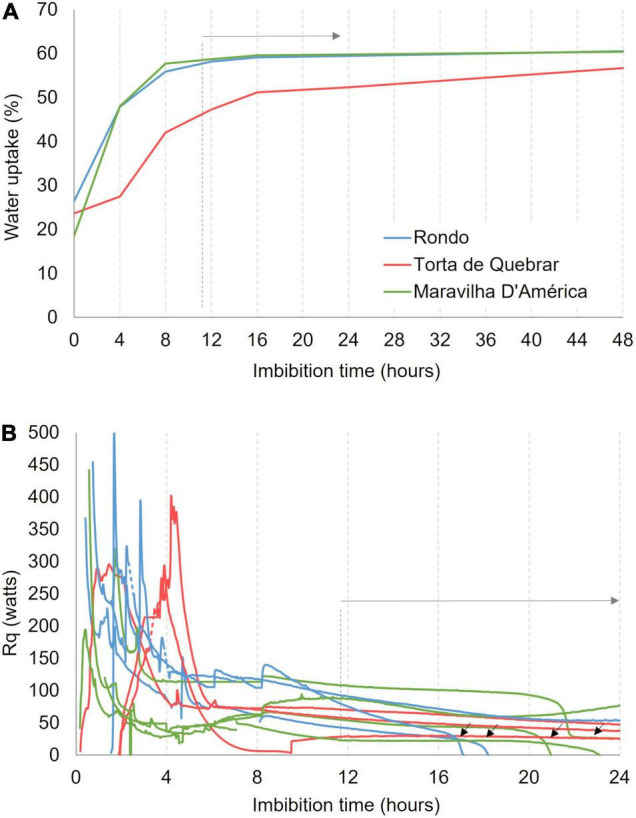
**(A)** Water uptake (WU) measured after 0, 4, 8, 12, 16, 24, and 48 h of imbibition in sterile tap water in three pea (*Pisum sativum* L.) cultivars (‘Rondo’, ‘Torta de Quebrar’, and ‘Maravilha d’América’) incubated at 25°C. The WU data are mean values of 30 seeds of each cultivar. **(B)** Heat rate (R*q*) emitted by single seeds of three pea cultivars (‘Rondo’, ‘Torta de Quebrar’, and ‘Maravilha d’América’) imbibed in sterile tap water, measured in isothermal mode at 25°C during 24 h.

The results of WU measurements at 25°C are shown in Figure 1A. The three cultivars started to absorb water immediately on soaking and produced a three-phase WU curve. At the beginning of the experiment, the cv. ‘Rondo’ (0.25 g) exhibited higher seed dry weight in comparison with cvs. ‘Torta de Quebrar’ (0.21 g) and ‘Maravilha d’América’ (0.18 g). Rapid WU, phase 1 or log phase, occurred during the first 12–16 h, depending on the cultivar. Phase 2 or stationary phase with little WU occurred between 16 and 24 h. The third phase, with a continuous increase in WU was more evident in cv. ‘Torta de Quebrar’ where it began at 16 h. The cvs. ‘Rondo’ and ‘Maravilha d’América’, attained the maximum WU value in the first 8 h, reaching the end of the log phase at 12 h with a WU of ∼58.2 and 58.6%, respectively. The cv. ‘Torta de Quebrar’ behaved differently, exhibiting a longer log phase and a lower WU value near 50% after the first 16 h. In cv. ‘Torta de Quebrar’, the stationary phase occured between 16 and 24 h with only a slight increase in WU from 51.2 to 52.3%. From 24 h until 48 h, WU slightly increased to reach the value of 56.7%.

Figure 1B shows the variability in R*q* during the first 12 h of imbibition when there was an exponential increase in WU. WU during this time period caused a large exothermic heat effect that occurs at slightly different times in individual seeds. These WU events were completed by 16 h and respiratory heat rates in viable seeds became constant for several hours. Calorespirometric measurements for determining phenotype viability during seed germination were therefore made after 16 h of imbibition. R_q_ measurements identified the inviable seeds of cvs. ‘Rondo’ and ‘Maravilha d’ América’ as indicated by the black arrows in Figure 1B. Note that these events were not caused by oxygen depletion since Thornton’s rule showed less than half the oxygen in the ampoule was respired during 24 h.

### Respiratory Properties of Seeds

**FIGURE 2:**
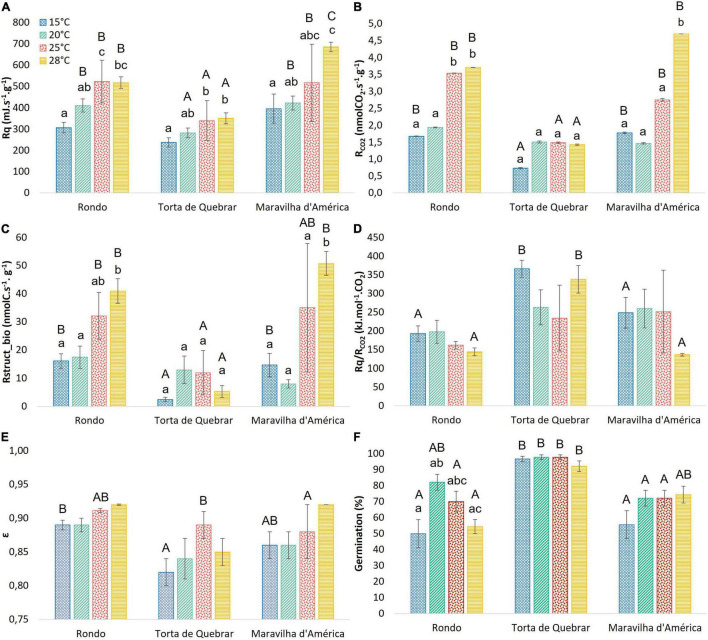
Calorespirometric parameters for three pea (*Pisum sativum* L.) cultivars (‘Rondo’, ‘Torta de Quebrar’, and ‘Maravilha d’América’) measured under isothermal mode at four different temperatures (15, 20, 25, and 28°C), 16 h post-imbibition in sterile tap water. **(A)** Respiratory heat rate—R*q*, **(B)** CO_2_ production rate—R_*CO2*_, **(C)** rate of growth of structural biomass—R_*struct_biomass*_, **(D)** the respiratory ratio—R*q*/R_*CO2*_, **(E)** the substrate carbon conversion efficiency—ε, and **(F)** final germination percentage achieved after 6 days of imbibition (data are mean values of 9 replicates of 10 seeds each, for a total of 90 seeds per temperature and cultivar). Data achieved for the calorespirometric parameters are the mean value of measurements taken from six seeds ± SE. Small letters indicate significant differences among temperatures for each cultivar, and capital letters indicate significant differences among cultivars at each temperature. Statistical significance was considered for *p* < 0.05 (small letters) and *p* < 0.01 (capital letters).

Figure 2 shows the R*q* was lower in cv. ‘Torta de Quebrar’ than in the other two cultivars at all temperatures. The R*q* values of cvs. ‘Rondo’ and ‘Maravilha d’América’ were essentially the same when compared at 15, 20, and 25°C, but differed significantly at 28°C. The R*q* values of all three cultivars increased with increasing temperature (Figure 2A). Carbon dioxide production rate (R_*CO2*_) and R_*struct_biomass*_ exhibited a similar pattern as that observed in R*q* (Figures 2B,C) with cv. ‘Torta de Quebrar’ lower than the other two cultivars. The calorespirometric ratio, R*q*/R_*CO2*_ was significantly higher in the cv. ‘Torta de Quebrar’ compared with the other two cultivars at 15 and 28°C (Figure 2D). The ε values were close to the same, between 0.8 and 0.9, for all three cultivars, but in ‘Torta de Quebrar’ it tended to be slightly lower than the other two cultivars, with significant differences at 15 and 25°C (Figure 2E).

The comparison of the seed lots of each cultivar revealed no significant differences in any of the calorespirometric parameters (as shown in the results of statistical analysis in Supplementary Table 1).

### Effect of Temperature on Respiratory Properties During Seed Germination

The effect of temperature on respiratory properties was evaluated in the pea seeds of the cultivars ‘Torta de Quebrar’, ‘Rondo’, and ‘Maravilha d’América’ after 16 h imbibition by running isothermal calorespirometric experiments at 15, 20, 25, and 28°C. Respiratory heat rate (R*q*) and RCO_2_ of cv. ‘Torta de Quebrar’ were notably less sensitive to temperature than in the other two cultivars. The temperature responses of R_*struct_biomass*_ and R*q*/R_*CO2*_ in ‘Torta de Quebrar’ also differed from the responses of these parameters in the other two cultivars (Figures 2C,D). Structural biomass formation rate, R_*struct_biomass*_ of ‘Torta de Quebrar’ showed a maximum between 20 and 25°C while the other cultivars showed increasing values above 20°C. The values of R*q*/R_*CO2*_ for ‘Torta de Quebrar’ showed a minimum between 20 and 25°C, in ‘Rondo’ showed a slight decrease with increasing temperature, and in ‘Maravilha d’ America’ was constant between 15–25°C and decreased at 28°C. The value of ε was invariant with temperature.

### Seed Germination During 6 Days of Imbibition

**FIGURE 3:**
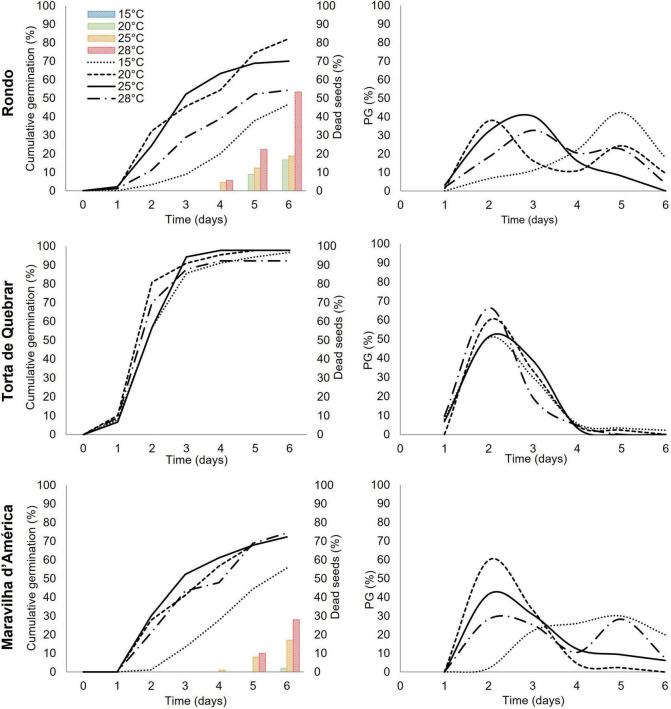
Cumulative germination, dead seeds per day, and germinated seeds per day (PG) at 15, 20, 25, and 28°C for three pea (*Pisum sativum* L.) cultivars (‘Rondo’, ‘Torta de Quebrar’, and ‘Maravilha d’América’) during 6 days post-imbibition in sterile tap water. Data were obtained from three independent experiments with 30 seeds per condition per cultivar (a total of 360 seeds per cultivar). The results of statistical analysis can be seen in Supplementary Table 2. The images of seeds during the 6 days of germination are shown in Supplementary Figure 2.

**FIGURE 4:**
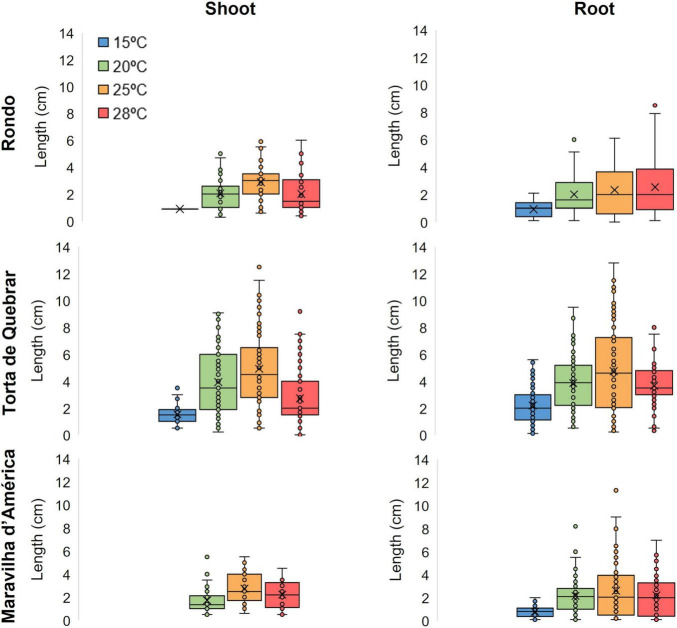
Shoot development and root elongation 6 days post-imbibition in sterile tap water in three different cultivars (‘Rondo’, ‘Torta de Quebrar’, and ‘Maravilha d’América’) incubated at four different temperatures: 15, 20, 25, and 28°C. Data were obtained from three independent experiments with 30 seeds per condition per cultivar (a total of 360 seeds per cultivar). The results of statistical analysis can be seen in Supplementary Tables 3, 4.

Three parameters, cumulative germination, fraction of seeds germinated per day (Figure 3), and root and shoot lengths (Figure 4), were monitored during 6 days of imbibition at four temperatures (15, 20, 25, and 28°C). Cumulative germination followed a sigmoid curve showing that seeds of the same cultivar did not germinate at the same time irrespective of thermal conditions. The germination time, the time required for onset, depended on both temperature and cultivar. The effect of temperature on germination was greater in cvs. ‘Rondo’ and ‘Maravilha d’América’ than in ‘Torta de Quebrar’. The decrease in germination at higher temperatures in cvs. ‘Rondo’ and ‘Maravilha d’América’ was associated with an increase in the number of dead seeds, associated with the development of mold growth, which reached the maximum at 28°C. No dead seeds were observed in any of the cultivars at 15°C.

Figure 4 shows root and shoot lengths measured after 6 days of imbibition at 15, 20, 25, and 28°C. Cultivars ‘Maravilha d’América’ and ‘Rondo’ exhibited a complete absence of shoots at 15°C. In cv. ‘Maravilha d’América’ at 20 and 25°C, less than 50% of the seeds exhibited shoot development (33.3 and 38.9%, respectively). The cv. ‘Torta de Quebrar’ exhibited the greatest root and shoot lengths and more than 70% of seeds with developed shoots at the three higher temperatures. High resilience under extreme temperatures, here associated with high seed vigor, was seen in cv. ‘Torta de Quebrar’, that exhibited seedling development at all tested temperatures.

The germination percentage achieved at 6 days post-imbibition is shown in Figure 2F. For cultivars ‘Torta de Quebrar’ and ‘Maravilha d’América’, no significant differences were observed in the percentage of germination at the four temperatures. For the cv. ‘Rondo’, the highest germination rate was achieved at 20 and 25°C, and the lowest germination rates were achieved at the extreme temperatures (15 and 28°C) (Figure 2F). In general, the cvs. ‘Rondo’ and ‘Maravilha d’América’ exhibited germination rates always lower than 90%, even at the optimum germination temperature. The cv. ‘Torta de Quebrar’ showed significantly higher germination rates at 15, 25, and 28°C in comparison with the other two cultivars, exhibiting almost 100% of the seeds germinated. At 20 and 28°C, cv. ‘Torta de Quebrar’ showed significantly higher germination rates in comparison with cv. ‘Maravilha d’América’ and cv. ‘Rondo’ (Figure 2F).

### Effect of Tegument on Seed Germination

**FIGURE 5:**
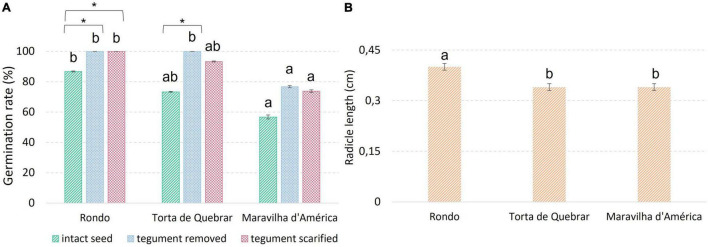
Parameters evaluated during a germination trial running at 25°C under dark conditions. **(A)** Total germination rate achieved 6 days after imbibition in intact seeds, in seeds devoid of tegument (removed 16 h after imbibition), and in scarified seeds. **(B)** Radicle length measured 16 h after imbibition in seeds of the three cultivars under study. Different letters indicate significant differences among cultivars. * Indicates significant differences among treatments in the same cultivar. Statistical significance was considered for *p* < 0.05 (*).

Embryo germination was assessed at 16 h after the imbibition period, when the radicle was not visible outside the tegument in any seed but it was possible to see the development if the tegument was removed. The removal of tegument improved the germination rate of all cultivars, reaching values of 100% in cv. ‘Rondo’ and ‘Torta de Quebrar’ (Figure 5). The results revealed a significantly higher radicle growth on cv. ‘Rondo’ than the other two cultivars. Physical inhibition was clearly visible on cv. ‘Rondo’ which showed an increase of 30% germination when tegument was removed or scarified.

### Correlation of Calorespirometric Parameters With Germination Parameters

In agreement with the results achieved by calorespirometry, the germination experiment performed with the three pea cultivars at the same temperatures tested in the calorimeter (15, 20, 25, and 28°C) revealed a strong effect of temperature vs. cultivar on the *aam* parameter (agronomically acceptable minimum) (Figure 3). In the same way, the minimum time required to achieve the *aam* was clearly dependent on the same interaction (temperature *vs.* cultivar) (Figure 3).

## Discussion

This study demonstrates for the first time the applicability of the calorespirometric method for phenotyping seed viability upon different temperatures. Although the most used seed phenotyping methods are based on the direct evaluation of seed germination capacity (viability) (FAO, 2013), new methodologies have recently been developed based on the measurement of seed metabolic activity, and in particular, on respiratory components or pathways [for review see Dalzielli and Tomlinson (2017)].

Reports on the use of calorespirometry in seeds are limited, the few reports available are mostly focused on the studies monitoring the germination process itself (Prat, 1952; Yamaguchi et al., 1990; Edelstein et al., 2001). The advantage of the calorespirometric method over the methods currently used for phenotyping seed viability is that in addition to measuring CO_2_ production rates, the production of metabolic heat is also measured, with both rates known to be correlated with plant growth rates (Criddle et al., 1991). Mohanapriya et al. (2019) highlighted the suitability of calorespirometry to assist pea seed breeding programs focused on the selection of inbred lines. However, the use of this technique to evaluate metabolic changes upon different growing temperatures was not considered.

The first step in developing a calorespirometric assay to screen seed viability at different temperatures is the determination of the best conditions to perform the measurements. A previous step of seed hydration is crucial to activate metabolism and achieve reliable measurements. When dry pea seeds were placed in the calorimeter, heat and CO_2_ production rates were below the detection limit of the equipment (data not shown), which is expected considering the low metabolic rate in dehydrated seeds (Walters et al., 2005; Bewley et al., 2013; Dalzielli and Tomlinson, 2017). In fact, it is known that seed imbibition immediately activates metabolism linked to germination, which is accompanied by an increase of respiration rates and heat production, the embryo being the most responsible for those metabolic changes (Domergue et al., 2019). Accordingly, an immediate increase in heat metabolic activity was detected in the calorimeter after seed imbibition (Figure 1A), which can be assigned to the activation of aerobic metabolism in early germination phase, also known as phase I (Weitbrecht et al., 2011). However, the instability of the measurements did not allow the determination of R_*CO2*_ during this phase. Therefore, an experiment was conducted to identify the most appropriate seed imbibition period that would provide the required stability of the metabolic heat and CO_2_ production rates during the first hours of seed germination. In addition, in a parallel experiment, seed WU was monitored during the first hours. These data showed that the increase on WU observed in the first hours of seed imbibition was accompanied by an immediate increase in metabolic heat rate in the seeds of the three cultivars. However, a few hours after imbibition, R*q* values strongly decreased. According to Bewley et al. (2013), the WU plateau phase is characterized by a decrease in respiration prior to germination. Nonetheless, differences were observed among cultivars. In cvs. ‘Maravilha d’América’ and ‘Rondo’, the stability of the heat rate was reached 8 h after imbibition, at the same time that the water absorption plateau phase was attained [phase II or middle phase according to Weitbrecht et al. (2011)], while the cv. ‘Torta de Quebrar’ exhibited a slower WU and reached the plateau at a later time (16 h after imbibition). This indicates that the duration of phase I is characteristic of the plant genotype, as previously shown by Dalzielli and Tomlinson (2017), which indicated a period that is comprised between 8 h in *Solanum lycopersicum* and 24 h in *Zea mays*.

Since it provides the required water to hydrate the cellular constituents, the imbibition period is crucial for seed germination, allowing the activation of cellular metabolism which includes the mobilization of food reserves and the activation of mitochondria and protein synthesis (Galland et al., 2014; Paszkiewicz et al., 2017). Although mitochondria activation has not yet been studied in pea seeds, we hypothesize that the required time to achieve stable and reproducible metabolic heat and CO_2_ rates is related to the mitochondria biogenesis processes and that by the end of this time, all mitochondria were functional, producing stable respiratory rates. Therefore, considering these results, the imbibition time selected for subsequent calorespirometric experiments was 16 h, for ensuring that all seed genotypes were in the WU plateau phase.

Once the seed imbibition period was selected, a trial was conducted to evaluate seed response at different temperatures. In this experiment, an effect of both the genotype and the temperature was observed on calorespirometric parameters R*q*, R_*CO2*_, and R_*struct_biomass*_. A genotype effect was also observed in the cultivars ‘Maravilha d’América’ and ‘Rondo’, which exhibited a faster WU than cv. ‘Torta de Quebrar’. The first two cultivars had low germination rates associated with dead seeds.

Temperature is one of the environmental factors with the greatest impact on plant growth and development. Previous reports have shown that plants adapt their growth to their environmental temperature by adapting their respiratory metabolism (Criddle et al., 2005). Although for cvs. ‘Torta de Quebrar’ and ‘Maravilha d’América’, no significant differences were observed in the percentage of germination between the four temperatures; for cv. ‘Rondo’, the highest germination was achieved at 20 and 25°C, which agrees with previous studies, where other authors observed that the optimum germination temperature was near 20°C in most cases (Raveneau et al., 2011).

Understanding the relationship between the measured and calculated calorespirometric parameters and the measured germination parameters requires the consideration of the metabolic processes underlying each of the calorespirometric parameters. Respiratory heat rate, R*q* is quantitatively related to the rates of O_2_ uptake and CO_2_ production by the following equation.


(6) Rq=470R=O2-ΔHRCO2-CO2ΔHRBstruct⁢_⁢biomass

Where R_*O2*_ is the rate of O_2_ uptake, ΔH_*CO2*_ is the enthalpy change for the production of a mole of CO_2_, R_*CO2*_ is the rate of CO_2_ production, ΔH_*B*_ is the enthalpy change for incorporation of a mole of carbon from the substrate into structural biomass, and R_*struct_biomass*_ is the rate of growth based on carbon. Respiratory heat rate, R*q* is thus a measure of the total rate of catabolic oxidation by both the alternative oxidase (AOX) and cytochrome oxidase (COX) pathways. Rearranging equation 6 gives R_*struct_biomass*_ in terms of the measured parameters, R*q* and R_*CO2*_.


(7) R=struct⁢_⁢biomass(-ΔHRCO2-CO2Rq)/ΔHB

The carbon use efficiency, ε, is then simply the ratio of the rate of carbon going into structural biomass to the rate of CO_2_ production.


(8) R/struct⁢_⁢biomassR=CO2ε/(1-ε)

The ratio, R*q*/R_*CO2*_, is related to ε by


(9) Rq/R=CO2-ΔH-CO2ΔH[ε/(1-ε)]B

Since ΔH_*CO2*_ and ΔH_*B*_ are constant and ΔH_*CO2*_ > > ΔH_*B*_, R*q*/R_*CO2*_ varies inversely with ε, i.e., R*q*/R_*CO2*_ decreases as ε increases.

The changes in environmental temperatures can strongly influence germination rates as an effect on membrane reorganization during imbibition (Fahad et al., 2017; Doria et al., 2019). In this experiment, the cv. ‘Torta de Quebrar’ had the most stable R*q* values across the temperature range from 15 to 28°C, and exhibited the highest values of seed germination, with slight variation across temperatures, revealing higher stability upon environmental temperature changes. Calorespirometry has usually been applied to predict growth responses as a function of temperature in various plant tissues (Nogales et al., 2013, 2015, 2020; Campos et al., 2016). One of the calorespirometric parameters that acts as a good indicator of the optimal temperature range for plant growth is the substrate carbon conversion efficiency (ε). According to previous studies, the value of ε varies between 0 and 0.85 in non-growing plants/tissues and rapidly growing vegetative plants and tissues, respectively (Amthor, 2000; Hansen et al., 2005). In the present research, ε values achieved in pea seeds were higher than those reported in the literature. These results may be related to the germination stage of seeds used which was not coincident with the germination stage of seeds used in available reports. Seeds were used in an early stage of germination, before the radicle protrusion, and no similar works are available in the literature for comparison. In fact, there are few calorespirometric studies at the level of metabolic changes under stress conditions in seeds. Nonetheless, cv. ‘Rondo’ was the cultivar that showed higher ε and R_*struct_biomass*_ values and lower germination rates for intact seeds of ‘Rondo’ initiated the strongest metabolic activity immediately after imbibition, mainly associated with embryo development which produced a larger embryo radicle in this cultivar in comparison with the other two cultivars. This larger length of embryo radicle at 16 h post-imbibition was not related with the germination rate, and no direct link between germination using intact seeds and the parameters recorded by calorespirometric measurements was detected. Our hypothesis to explain this discrepancy in the results is based on the putative effect of physical inhibition on cv. ‘Rondo’. To elucidate this effect, a germination trial was established considering the scarification of seeds and the complete removal of the tegument, and a different result was achieved in the germination rate in seeds devoid of the tegument. The use of seeds after removing their tegument revealed 100% germination rates in cv. ‘Rondo’ and a radicle length significantly higher on this cultivar in comparison with the other two. In the *Fabaceae* family, seed dormancy is currently observed (Smıkal et al., 2014) and has been mainly attributed to the physical dormancy (Martins et al., 2012), which was clearly seen in cv. ‘Rondo’. The tegument thickness was highlighted as the main factor responsible for the physical dormancy and the consequent inhibition of seed germination in wild pea (*P. sativum* subsp. *elatius*) (Hradilová et al., 2019).

Carbon use efficiency is calculated from the R*q*/R_*CO2*_ ratio, which is informative of the activity of catabolic and anabolic metabolism. Stressful conditions, due for example, to extreme temperatures, lead to the changes in metabolic pathways, which are reflected in high values of R*q*/R_*CO2*_ and low values of ε (Rank et al., 1991; Hansen et al., 2009). The model that relates these two calorespirometric parameters assumes that the temperature limits of plant growth correspond to the genetically determined biochemical limits. When a plant’s environment reaches these temperatures, the efficiency of respiration goes to zero (McCarlie et al., 2003). In this work, comparisons made across the four temperatures for each cultivar showed stable values over the studied temperature range. However, the comparison among cultivars revealed the lower values of ε for the cv. ‘Torta de Quebrar’ than for the other two cultivars at 15, 25, and 28°C, a relation more clearly seen in R*q*/R_*CO2*_, which was significantly higher in cv. ‘Torta de Quebrar’ than in the other two cultivars at 15 and 28°C. The lower ε and higher R*q*/R_*CO2*_ values were inversely correlated with germination rates, as cv. ‘Torta de Quebrar’ showed a high germination rate at all temperatures tested. Taken together, these results lead to the conclusion that the cv. ‘Torta de Quebrar’ is more vigorous in comparison to the other two cultivars. A greater engagement of stress-responsive genes, in particular genes involved in the control of reactive oxygen species (ROS) homeostasis, which includes the enzymes of ROS-scavenging mechanisms and the non-enzymatic mechanism associated with antioxidant metabolites, could be hypothesized as responsible of the increased resilient behavior.

Several studies have shown that in green tissues, the biomass growth rate is correlated with the *R*_*struct_biomass*_ calculated from calorespirometric parameters (McCarlie et al., 2003; Yu et al., 2008). Nogales et al. (2015), for example, demonstrated the correlation between biomass growth and *R*_*struct_biomass*_ in taproot secondary meristems, but only at a more advanced stage of development. This work examined the relationship between the *R*_*struct_biomass*_ parameter and the percentage of germination in the three pea cultivars under study. While *R*_*struct_biomass*_ remained stable across the temperature range from 15 to 28°C in cv. ‘Torta de Quebrar’, in the cvs. ‘Rondo’ and the ‘Maravilha d’América’, an increase was observed as temperature increased. Germination rates thus followed a different pattern, the lower values of *R*_*struct_biomass*_ and ε and higher values of R*q*/R_*CO2*_ corresponded to higher germination rates in the experiment using intact seeds. The experiment made with seeds devoid of tegument clarified the direct proportionality between germination and ε and *R*_*struct_biomass*_. Explaining the inverse pattern found for seed germination requires understanding that there are two sources of CO_2_ and two kinds of inefficiencies in respiratory metabolism in plants. CO_2_ is produced by direct oxidation of substrate by O_2_ in catabolism and by reductive elimination in anabolism. The rate of CO_2_ production by direct oxidation is quantified by 470R*q* and the rate of production of CO_2_ by reductive elimination is quantified by the function −ΔH_*B*_*R*_*struct_biomass*_, ΔH_*B*_ is typically +30 kJ/mole in growing leaf tissues (Ellingson et al., 2003). In the model used here, *R*_*struct_biomass*_ is equated to the rate of CO_2_ production by reductive elimination divided by ΔH_*B*_. Since none of these parameters are likely to differ between seeds and other growing plant tissues, we must look to the inefficiencies in respiratory metabolism, decreased efficiency in producing ATP in catabolism, and the futile hydrolysis of ATP in anabolism. Although the futile hydrolysis of ATP is a possible explanation, the extensive literature on the activation of alternative oxidase pathway during seed germination and stress strongly suggests that this is the cause of the inverse relation between the calculated *R*_*struct_biomass*_ and ε values and germination and stress response (Figure 2). The link between the alternative oxidase (AOX), the key enzyme in the alternative respiratory pathway, and structural biomass formation was previously highlighted by Campos et al. (2016) in a carrot primary culture system. The authors reported an increase in *AOX* gene expression associated with a peak in *R*_*struct_biomass*_. Further research aiming to evaluate the involvement of AOX in pea seed germination upon different temperatures will be considered to elucidate the role of ROS-homeostasis pathway and efficient germination.

## Conclusion

The results obtained by calorespirometry provide important knowledge about seed metabolism during the complex process of pea seed germination, revealing the potential existence of physical inhibition. Considering the existence of an inhibitor effect associated with tegument, the data are strongly linked to seed viability which allows us to propose that calorespirometry is a useful tool for phenotyping physiologic efficiency at different temperatures during early germination stages. Moreover, it could identify the seeds with the highest resilience to temperature variation, which were in this case ‘Torta de Quebrar’. This cultivar showed the highest and most consistent germination at all temperatures when compared with the other two cultivars. ‘Torta de Quebrar’ was also distinguished from the other two cultivars by having the lowest values of R*q*, R_*CO2*_, R_*struct_biomass*_, and ε and the highest values of R*q*/R_*CO2*_. ‘Torta de Quebrar’ also showed the smallest temperature dependence of R*q*, R_*CO2*_, and R_*struct_biomass*_ compared to the other two cultivars. All these differences among cultivars and the temperature responses are consistent with a putative greater engagement of the AOX pathway relative to the COX pathway in ‘Torta de Quebrar’ compared with the other cultivars. The lower temperature dependence of the calorespirometric parameters suggests a greater increase in the AOX/COX ratio with increasing temperature in ‘Torta de Quebrar’ than in the other cultivars. Thus, we can conclude that the superior germination of ‘Torta de Quebrar’ was a consequence of greater engagement of the AOX pathway. This conclusion agrees with the literature consensus that the AOX pathway is a means of relieving stress from temperature and drought (Wang and Vanlerberghe, 2013; Velada et al., 2014; Dahal and Vanlerberghe, 2017; Campos et al., 2021). Nevertheless, further studies will be considered to evaluate the involvement of AOX on pea seed germination including pea genotypes with differences in calorespirometric measurements.

## Data Availability Statement

The raw data supporting the conclusions of this article will be made available by the authors, without undue reservation.

## Author Contributions

HC, AN, and LR contributed to the conception and design of the study. LR, FS, and AR set up the germination trials. HC and LR performed calorespirometric analyses and wrote the first draft of the manuscript. LR, AN, and LH performed statistical data analysis. AN and LH reviewed the first draft of the manuscript. HC reviewed and finalized the manuscript and warranted financial support. All authors contributed to manuscript revision, read, and approved the submitted version.

## Author Disclaimer

The opinions expressed and arguments employed herein do not necessarily reflect the official views of the EC and the Swiss government. Neither the European Commission/SERI nor any person acting on behalf of the Commission/SERI is responsible for the use which might be made of the information provided on this publication.

## Conflict of Interest

The authors declare that the research was conducted in the absence of any commercial or financial relationships that could be construed as a potential conflict of interest.

## Publisher’s Note

All claims expressed in this article are solely those of the authors and do not necessarily represent those of their affiliated organizations, or those of the publisher, the editors and the reviewers. Any product that may be evaluated in this article, or claim that may be made by its manufacturer, is not guaranteed or endorsed by the publisher.
