# Supplementary material for: Exploring the Applicability of Calorespirometry to Assess Seed Metabolic Stability Upon Temperature Stress Conditions—Pisum sativum L. Used as a Case Study
Source: Front Plant Sci. 2022 Apr 27;13:827117. doi: 10.3389/fpls.2022.827117 (PMC9094064; doi:10.3389/fpls.2022.827117)
Supplement: Supplementary file 1 [file Data_Sheet_1.docx]

Supplementary Material


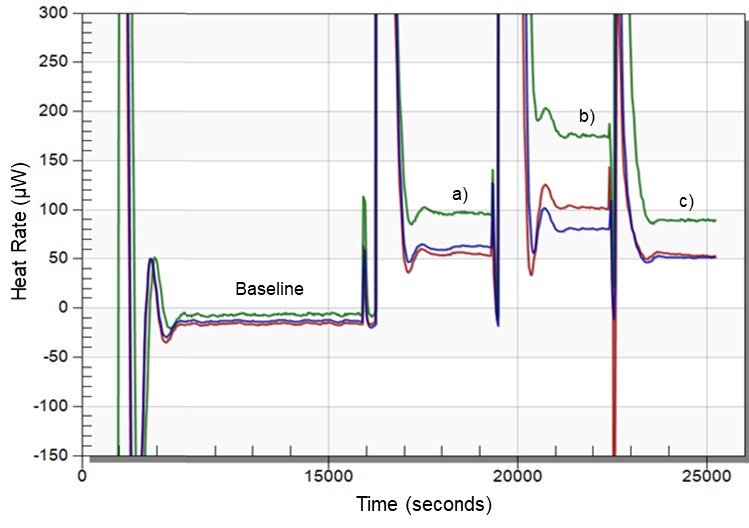


**Supplementary Figure S1.** Raw data collected at 25°C on the three cultivars simultaneously (green line corresponds to cv. ‘Maravilha d’América’, blue line to cv. ‘Torta de Quebrar’ and red line to cv. ‘Rondo’). a) initial heat rate collected from each seed; b) heat rate recorded in the presence of a vial containing 50µl of 0.4 M NaOH solution; c) heat rate measured after removal of the vial containing the NaOH from the ampoules.

**
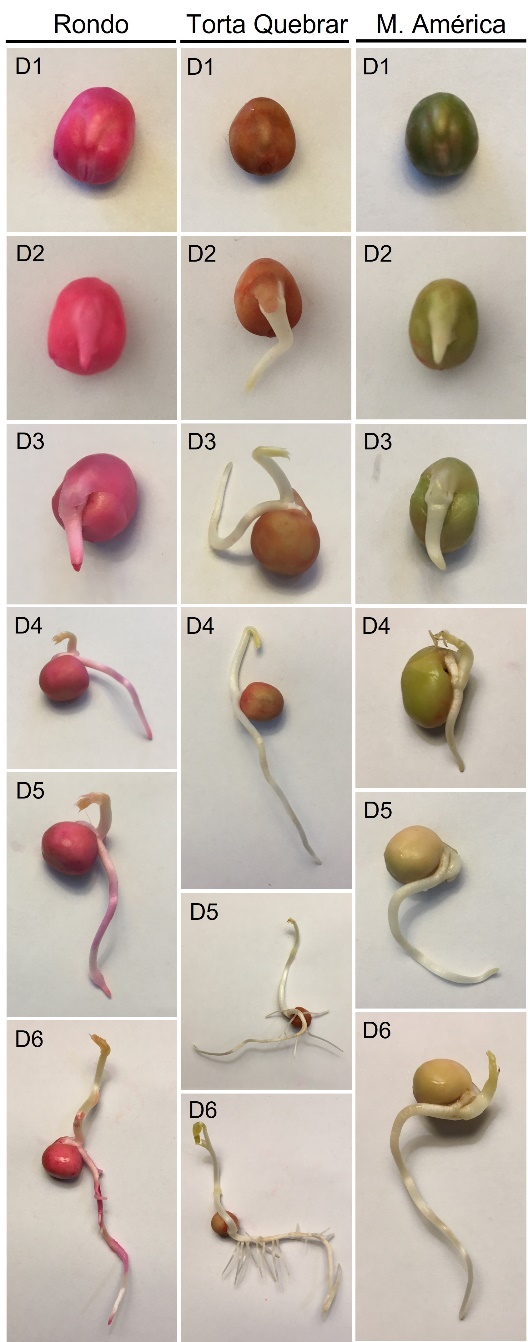
**

**Supplementary Figure S2.** Illustrative images of the evolution of pea seed germination over six days from three different cultivars (cvs. ‘Rondo’, ‘Torta de Quebrar’ and ‘Maravilha d’América’). After six days of imbibition the aerial and radicular systems are well developed. The principal root is composed of several secondary roots, and the apical shoot exhibits the primary photosynthetically active leaves (green colored). D1 – 1 day after imbibition, D2, D3, D4, D5 and D6 – 2, 3, 4, 5 and 6 days after imbibition.

**Supplementary Table S1.** Comparison of calorespirometric parameters of two different lots (1 and 2) for three pea (*Pisum sativum* L.) cultivars (‘Rondo’, ‘Torta de Quebrar’ and ‘Maravilha d’América’) measured under isothermal mode at 25°C, 16 h post imbibition in sterile tap water. Respiratory heat rate - Rq, CO_2_ production rate - R_CO2_, rate of growth of structural biomass - R_struct_biomass_, the respiratory ratio - Rq/R_CO2_, and the substrate carbon conversion efficiency - ε. Data are the mean value of measurements ± standard error. Mean comparisons were performed between both lots for each cultivar. Statistical significance was considered for P<0.05.

|  |  | Rq | | R_CO2_ | | R_struct_biomass_ | | Rq/R_CO2_ | | ε | |
| --- | --- | --- | --- | --- | --- | --- | --- | --- | --- | --- | --- |
| CV | Lot | Mean (%) ± SE | *P* | Mean (%) ± SE | *P* | Mean (%) ± SE | *P* | Mean (%) ± SE | *P* | Mean (%) ± SE | *P* |
| Rondo | 1 | 565.5 ± 32.3 | *0.10* | 3.5 ± 0.5 | *0.1* | 32.9 ± 4.0 | *0.1* | 161.9 ± 4.0 | *0.11* | 0.91 ± 0.0 | *0.07* |
|  | 2 | 634.7 ± 13.8 |  | 4.8 ± 0.5 |  | 52.6 ± 7.5 |  | 135.3 ± 12.1 |  | 0.92 ± 0.0 |  |
| Torta de Quebrar | 1 | 340.1 ± 9.9 | *0.40* | 1.3 ± 0.1 | *0.13* | 11.9 ± +3.2 | *0.38* | 234.4 ± 33.3 | *0.16* | 0.89 ± 0.0 | *0.35* |
|  | 2 | 361.2 ± 17.9 |  | 1.82 ± 0.2 |  | 16.5 ± 3.4 |  | 181.2 ± 16.4 |  | 0.90 ± 0.0 |  |
| Maravilha d’América | 1 | 517.2 ± 75.6 | *0.16* | 2.7 ± 0.7 | *0.21* | 35.1 ± 10.2 | *0.43* | 221.3 ± 34.1 | *0.06* | 0.88 ± 0.02 | *0.08* |
|  | 2 | 628.2 ± 29.1 |  | 3.9 ± 0.5 |  | 48.3 ± 9.8 |  | 149.6 ± 18.1 |  | 0.91 ± 0.0 |  |

**Supplementary Table S2.** Cumulative germination achieved at 15, 20, 25 and 28°C for three pea (*Pisum sativum* L.) cultivars (‘Rondo’, ‘Torta de Quebrar’ and ‘Maravilha d’América’) during six days. Data were obtained from three independent experiments with thirty seeds per condition and per cultivar (a total of 360 seeds per cultivar). Data are the mean value of measurements ± standard error. Mean comparisons were performed among temperatures for each cultivar. Statistical significance was considered for P<0.05. Different letters indicate significant differences among temperatures.

|  |  | **Days of germination** | | | | | | | | | | | | | | | |
| --- | --- | --- | --- | --- | --- | --- | --- | --- | --- | --- | --- | --- | --- | --- | --- | --- | --- |
|  |  | 1 | | 2 | | 3 | | | 4 | | | 5 | | | 6 | | |
| CV | T (ºC) | Mean (%) ± SE | *P* | Mean (%) ± SE | *P* | Mean (%) ± SE | | *P* | Mean (%) ± SE | | *P* | Mean (%) ± SE | | *P* | Mean (%) ± SE | | *P* |
| Rondo | 15 | 0 ± 0 | *0.53* | 3.3 ± 2.4 ^a^ | *0.04* | 8.9 ± 3.9 ^a^ | *0.01* | | 20 ± 4.7 ^a^ | *0.01* | | 37.8 ± 7.3 ^a^ | *0.003* | | 46.7 ± 8.8 ^a^ | *0.004* | |
|  | 20 | 1.1 ± 1.1 |  | 32.2 ± 10.2 ^b^ |  | 45.6 ± 10 ^b^ |  |  | 54.4 ± 11.8 ^b^ |  |  | 74.4 ± 6.3 ^b^ |  |  | 82.2 ± 4.9 ^b^ |  |  |
|  | 25 | 2.2 ± 1.5 |  | 24.4 ± 8.7 ^b^ |  | 52.2 ± 5.5 ^b^ |  |  | 63.3 ± 7.3 ^b^ |  |  | 68.9 ± 6.2 ^b^ |  |  | 70 ± 6.4 ^ab^ |  |  |
|  | 28 | 1.1 ± 1.1 |  | 11.1 ± 3.9 ^ab^ |  | 28.9 ± 7.3 ^ab^ |  |  | 38.9 ± 6.1 ^ab^ |  |  | 52.2 ± 4.9 ^ab^ |  |  | 54.4 ± 4.4 ^ac^ |  |  |
| Torta de Quebrar | 15 | 7.8 ± 5.2 | *0.93* | 56.7 ± 5.8 | *0.15* | 85.6 ± +4.4 | *0.79* | | 91.1 ± 2.6 | *0.42* | | 94.4 ± 1.7 | *0.35* | | 96.7 ± 1.7 | *0.34* | |
|  | 20 | 10.1 ± 5.3 |  | 80.9 ± 9.0 |  | 91.0 ± 4.4 |  |  | 95.5 ± 2.4 |  |  | 97.8 ± 1.1 |  |  | 97.8 ± 1.5 |  |  |
|  | 25 | 6.7 ± 3.3 |  | 56.7 ± 14.6 |  | 94.4 ± 2.9 |  |  | 97.8 ± 1.1 |  |  | 97.8 ± 1.5 |  |  | 97.8 ± 1.5 |  |  |
|  | 28 | 8.9 ± 3.9 |  | 70.0 ± 5.8 |  | 87.9 ± 3.2 |  |  | 92.2 ± 3.2 |  |  | 92.2 ± 3.2 |  |  | 92.2 ± 3.2 |  |  |
| Maravilha d’América | 15 | 0 ± 0 | *1* | 1.10 ± 1.10 ^a^ | *0.01* | 13.3 ± 4.4 ^a^ | *0.002* | | 27.8 ± 4.9 ^a^ | *0.01* | | 44.0 ± 5.8 ^a^ | *0.01* | | 55.6 ± 8.7 | *0.11* | |
|  | 20 | 0 ± 0 |  | 27.8 ± 4.0 ^b^ |  | 41.1 ± 6.9 ^b^ |  |  | 56.7 ± 7.1 ^b^ |  |  | 67.8 ± 5.4 ^b^ |  |  | 72.2 ± 4.9 |  |  |
|  | 25 | 0 ± 0 |  | 30 ± 9.8 ^b^ |  | 52.2 ± 6.6 ^b^ |  |  | 61.1 ± 6.7 ^b^ |  |  | 67.8 ± 5.7 ^b^ |  |  | 72.2 ± 4.4 |  |  |
|  | 28 | 0 ± 0 |  | 21.1 ± 5.4 ^b^ |  | 43.3 ± 10.2 ^b^ |  |  | 47.8 ± 5.9 ^ab^ |  |  | 68.9 ± 5.8 ^b^ |  |  | 74.4 ± 5.3 |  |  |

CV: cultivar; T:temperature; SE: Standard error; P: statistical significance.

**Supplementary Table S3.** Shoot development and root elongation measured six days post imbibition in sterile tap water in three different cultivars (‘Rondo’, ‘Torta de Quebrar’ and ‘Maravilha d’América’) compared among four different incubation temperatures: 15, 20, 25 and 28°C. Data were obtained from three independent experiments with thirty seeds per condition per cultivar (a total of 360 seeds per cultivar). Data are the mean value of measurements ± standard error. Mean comparisons were performed among temperatures for each cultivar. Statistical significance was considered for P<0.05. Different letters indicate significant differences among temperatures.

|  | **Shoot development (cm)** | | | | | | **Root elongation (cm)** | | | | | |
| --- | --- | --- | --- | --- | --- | --- | --- | --- | --- | --- | --- | --- |
|  | Rondo | | Torta de Quebrar | | Maravilha d´América | | Rondo | | Torta de Quebrar | | Maravilha d´América | |
| T (°C) | Mean (%) ± SE | *P* | Mean (%) ± SE | *P* | Mean (%) ± SE | *P* | Mean (%) ± SE | *P* | Mean (%) ± SE | *P* | Mean (%) ± SE | *P* |
| 15 | 0 ± 0 ^a^ | *0.00* | 0.26 ± 0.13 ^a^ | *0.00* | 0 ± 0 ^a^ | *0.00* | 0.17 ± 0.07 ^a^ | *0.00* | 1.54 ± 0.27 ^a^ | *0.00* | 0.14 ± 0.05 ^a^ | *0.00* |
| 20 | 0.67 ± 0.23 ^b^ |  | 2.61 ± 0.41 ^b^ |  | 0.75 ± 0.26 ^b^ |  | 1.45 ± 0.33 ^b^ |  | 3.54 ± 0.39 ^b^ |  | 1.68 ± 0.37 ^b^ |  |
| 25 | 1.72 ± 0.29 ^cd^ |  | 2.40 ± 0.44 ^b^ |  | 1.40 ± 0.24 ^cd^ |  | 2.12 ± 0.26 ^b^ |  | 2.94 ± 0.33 ^b^ |  | 1.84 ± 0.29 ^b^ |  |
| 28 | 0.89 ± 0.13 ^bd^ |  | 2.17 ± 0.21 ^b^ |  | 0.73 ± 0.26 ^b^ |  | 1.84 ± 0.46 ^b^ |  | 3.92 ± 0.40 ^b^ |  | 1.70 ± 0.36 ^b^ |  |

**Supplementary Table S4.** Shoot and root elongation measured six days post imbibition in sterile tap water incubated at four different temperatures: 15, 20, 25 and 28°C) compared among three different cultivars (‘Rondo’, ‘Torta de Quebrar’ and ‘Maravilha d’América’). Data were obtained from three independent experiments with thirty seeds per condition per cultivar (a total of 360 seeds per cultivar). Data are the mean value of measurements ± standard error. Mean comparisons were performed among temperatures for each cultivar. Statistical significance was considered for P<0.05. Different letters indicate significant differences among cultivars.

|  | **Shoot development (cm)** | | | | | | | | **Root elongation (cm)** | | | | | | | |
| --- | --- | --- | --- | --- | --- | --- | --- | --- | --- | --- | --- | --- | --- | --- | --- | --- |
|  | **15ºC** | | **20 ºC** | | **25 ºC** | | **28 ºC** | | **15 ºC** | | **20 ºC** | | **25 ºC** | | **28 ºC** | |
| CV. | Mean (%) ± SE | *P* | Mean (%) ± SE | *P* | Mean (%) ± SE | *P* | Mean (%) ± SE | *P* | Mean (%) ± SE | *P* | Mean (%) ± SE | *P* | Mean (%) ± SE | *P* | Mean (%) ±  SE | *P* |
| RD | 0.0 ± 0.0 ^a^ | *0.01* | 0.67 ± 0.23 ^a^ | *0.00* | 1.72 ± 0.29 | *0.15* | 0.89 ± 0.13^a^ | 0.00 | 0.17 ± 0.07^a^ | *0.00* | 1.45 ± 0.33^a^ | *0.00* | 2.12 ± 0.26^ab^ | *0.05* | 1.84 ± 0.46 ^a^ | *0.00* |
| TQ | 0.26 ± 0.13^b^ |  | 2.61 ± 0.41 ^b^ |  | 2.40 ± 0.44 |  | 2.17 ± 0.21^b^ |  | 1.54 ± 0.27^b^ |  | 3.54 ± 0.39^b^ |  | 2.94 ± 0.33^b^ |  | 3.92 ± 0.40 ^b^ |  |
| MA | 0.0 ± 0.0 ^a^ |  | 0.75 ± 0.26 ^a^ |  | 1.40 ± 0.24 |  | 0.73 ± 0.26^a^ |  | 0.14 ± 0.05^a^ |  | 1.68 ± 0.37^a^ |  | 1.84 ± 0.29^a^ |  | 1.70 ± 0.36 ^a^ |  |
